# Genetic Susceptibility to Rotavirus Gastroenteritis and Vaccine Effectiveness in Taiwanese Children

**DOI:** 10.1038/s41598-017-06686-y

**Published:** 2017-07-25

**Authors:** Ting-An Yang, Ju-Yin Hou, Yhu-Chering Huang, Chih-Jung Chen

**Affiliations:** 1 College of Medicine, Chang Gung University, 333 Taoyuan, Taiwan; 2 Division of Paediatric Infectious Diseases, Department of Paediatrics, Chang Gung Memorial Hospital, 333 Taoyuan, Taiwan

## Abstract

The genetic susceptibility to and vaccine effectiveness against rotavirus gastroenteritis were different in distinct ethnic groups. The case-control study was aimed to evaluate the effectiveness of rotavirus vaccines and associations between the histo-blood group antigens and susceptibility to rotavirus infections in a Taiwanese population. Cases were children <18 years old who were hospitalized because of laboratory-confirmed rotavirus infection. Controls were healthy children matched to cases by age and gender. The secretor status and Lewis antigen and ABO types were determined by molecular methods. A total of 68 cases and 133 controls were included. Rotavirus immunization was recorded in 8 (12%) cases and 77 (58%) controls, indicating a vaccine effectiveness of 90.3% (95% confidence interval [CI], 78.1% − 95.7%). The secretor and Lewis-positive genotypes were independently associated with increased risk of rotavirus infections (matched odds ratio [mOR] 28.5, 95% CI 2.94–277, *P* = 0.003 and mOR 16.8, 95% CI 1.08–2601, *P* = 0.04, respectively). The distribution of ABO blood types did not differ significantly between cases and controls (*P* = 0.47). In conclusion, Taiwanese children with the secretor genotype and Lewis-positive genotype were at increased risk of moderate-to-severe rotavirus infections. The illness can be effectively prevented by immunization in this population.

## Introduction

Rotavirus was the leading cause of acute gastroenteritis (AGE) and accounted for 20% to 50% of pediatric hospitalizations for acute diarrhea before the wide use of effective vaccines^1, 2^. A dual classification system has been used for typing rotavirus based on two viral surface proteins. Of them, glycoprotein VP7 defines the G serotype, and the protease-sensitive protein VP4 defines the P serotype^3^. VP4 can further be modified by trypsin into VP5* and VP8*^4^. It has been proposed that the spike protein VP8* can recognize histo-blood group antigens (HBGAs) of hosts to mediate cell attachment^5^. The HBGA contains a carbohydrate structure, namely, H type 1 antigen. Its synthesis is dependent on the α1,2-fucosyltransferase encoded by the *FUT2* gene, which determines the secretor status. In addition, there are two other enzymes, the α1,3/4-fucosyltransferase encoded by the *FUT3* gene (determining the Lewis antigen) and the A or B enzyme encoded by the *ABO* gene, involved in the biosynthesis of HBGA. The three alleles together are the potential determinants of host susceptibility to a variety of enteropathogens, including rotavirus.

The essential role of HBGA in rotavirus infection has been demonstrated in recent studies. A study in France showed that none of the rotavirus P[8]-infected cases were non-secretors, while 20% of the control population were non-secretors^6^. Another study in Burkina Faso and Nicaragua demonstrated that all P[8]-infected children were positive for Lewis and secretor antigens^7^. One systematic review including 4 publications has further demonstrated that secretors are more susceptible to P[8] infection than are non secretors^8^. However, the distribution of HBGA genotypes is largely different in distinct ethnic groups. For instance, there are very few ‘non-secretors’ among Southeast and East Asians^9–11^. Furthermore, the circulating rotavirus strains can change after the wide use of vaccines, which may compromise the effectiveness of immunization. It remains essential to continuously monitor vaccine effectiveness and determine how children are susceptible to rotavirus infections during or after implementation of rotavirus vaccination. To this end, we conducted a case-control study to explore the associations between host genetic factors, including secretor status, Lewis antigen and ABO blood types and severity of AGE caused by rotavirus in Taiwanese children. The vaccine effectiveness during the study period was also assessed.

## Results

### Features of the study subjects and effectiveness of the rotavirus vaccines

**Figure 1:**
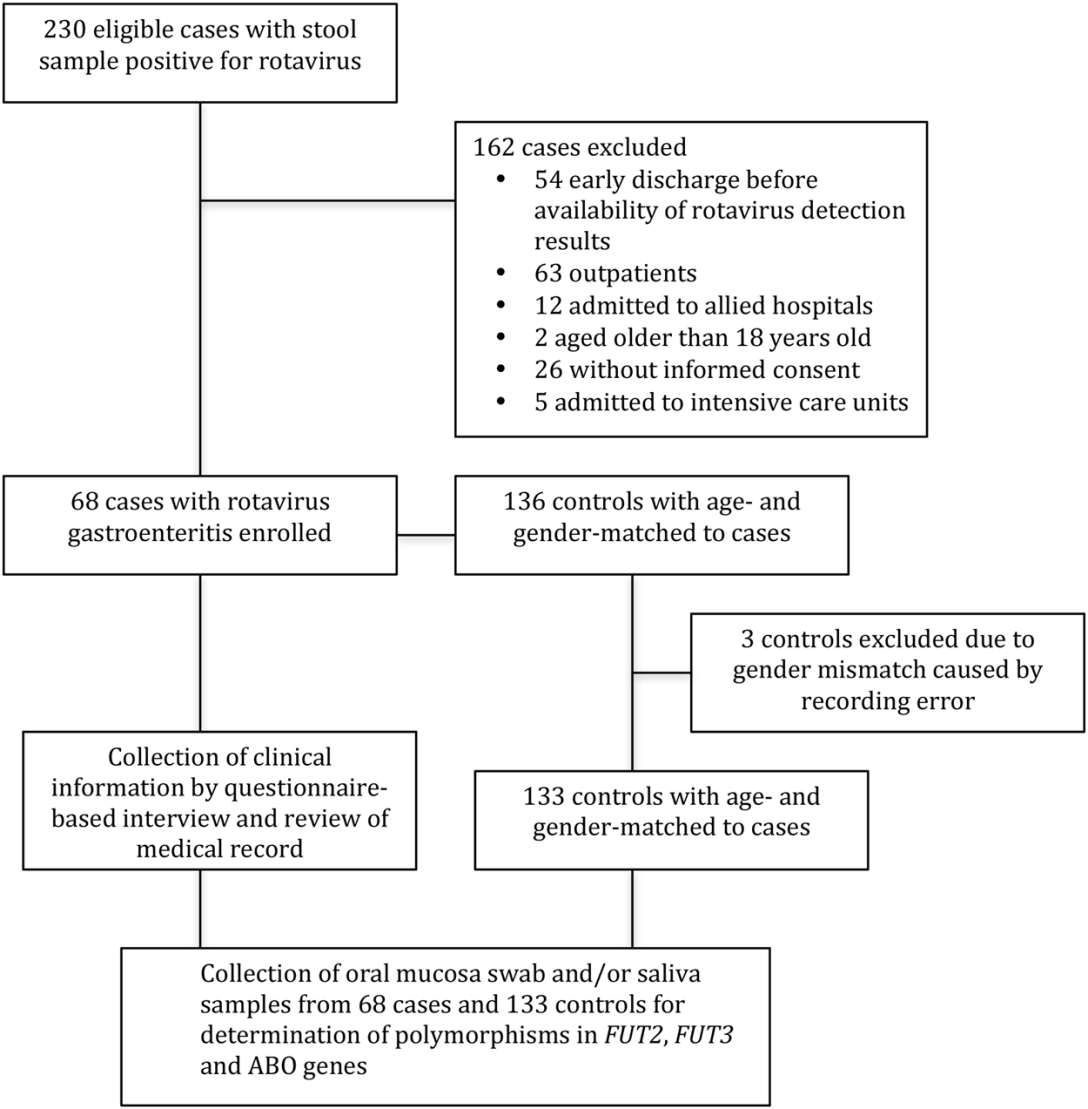
Flow chart depicting the recruitment of the case and control subjects.

During the study period, 230 eligible patients with laboratory-confirmed rotavirus infection were identified. Of them, 68 (29.6%) cases were enrolled in this study. The reasons for exclusion of the remaining 162 cases are shown in Fig. 1. Among the 68 enrolled cases, 36 were male, and the mean age was 42.6 ± 31.6 months (median, 33.8 months, range, 1.6–135.0 months). Fifty-five (80.9%) cases were categorized as severe AGE, as defined with a Vesikari score > 10, whereas the other 13 cases (19.1%) were categorized as moderate AGE (Vesikari score 7–10). In total, 136 controls 2:1 matched to 68 cases by age and gender were initially enrolled. Unfortunately, three controls with a recording error in gender were identified. The controls with a gender mismatch were excluded, and the final analysis included 68 cases and 133 controls (Supplementary Table 1). Any dose of rotavirus immunization was recorded in 8 (12%) cases and 77 (58%) controls. The effectiveness of the vaccine against rotavirus infections was 90.3% (95% confidence interval [CI], 78.1% − 95.7%) in this cohort. The detailed demographics of all study subjects and the clinical features of the case subjects of distinct disease severity are displayed in Supplemental Table 1.

### Distributions of secretor status, Lewis genotypes and ABO types in cases and controls

The detailed distribution of secretor status, Lewis genotypes and ABO blood types among the case subjects with distinct severity and control subjects are displayed in Table 1. Four SNPs in *FUT2* alleles were identified in this study, including a silent mutation (C357T), a missense mutation (A385T) and two nonsense mutations (C571T and G849A). All of the cases and controls had either a secretor or weak-secretor genotype without any non-secretors in this study. Most of the secretors carried alleles with the C357T synonymous mutation (84.7%, 144/170). All of the weak secretors had the A385T missense mutation. Of the 68 rotavirus-positive cases, 67 (99%) were characterized as secretors, whereas 101 (76%) of the 133 controls were secretors (*P* < 0.001), suggesting that secretor status is a risk factor for rotavirus infection. Consistent with this finding, the multivariate analysis further disclosed that the secretors were associated with a 28.6-fold increased risk of rotavirus infection compared with weak secretors (*P* = 0.004, Table 2).

**Table 1:** Distribution of secretor status (*FUT2*), Lewis genotype (*FUT3*) and ABO types among cases with rotavirus gastroenteritis of distinct severities and healthy control subjects.

HBGAs genotype	Cases			Controls N = 133 (%)	*P*_1_^*^	*P*_2_^*^
	All N = 68 (%)	Moderate N = 13 (%)	Severe N = 55 (%)			
*FUT2*					0.62	<0.001
Secretor genotype^$^	67 (99)	13 (100)	54 (98)	101 (76)		
Se/Se	0 (0)	0 (0)	0 (0)	0 (0)		
Se/C357T	11(16)	2 (15)	9 (17)	9 (6.7)		
Se/A385T	10 (15)	2 (15)	8 (15)	15 (11)		
Se/C571T	0 (0)	0 (0)	1 (1.0)	1 (0.8)		
C357T/C357T	15 (22)	3 (23)	12 (22)	20 (15)		
C357T/A385T	29 (43)	6 (46)	23 (42)	52 (39)		
C357T/C571T	1 (1.5)	0 (0)	1 (1.8)	3 (2.3)		
C357T/G849A	1 (1.5)	0 (0)	1 (1.8)	1 (0.8)		
Weak-secretor genotype^$^	1 (1.5)	0 (0)	1 (1.8)	32 (24)		
A385T/A385T	0 (0)	0 (0)	0 (0)	29 (22)		
A385T/C571T	1 (1.5)	0 (0)	1 (1.8)	1 (0.8)		
A385T/G849A	0 (0)	0 (0)	0 (0)	2 (1.5)		
*FUT3*					0.62	0.03
Lewis-positive genotype^#^	67 (99)	13 (100)	54 (98)	120 (90)		
Le/Le	42 (62)	9 (69)	33 (60)	56 (42)		
Le/G508A	17 (25)	3 (23)	14 (26)	25 (19)		
Le/T1067A	8 (12)	1 (7.7)	7 (13)	39 (29)		
Lewis-negative genotype^#^	1 (1.5)	0 (0)	1 (1.8)	13 (9.8)		
G508A/T1067A	1 (1.5)	0 (0)	1 (1.8)	13 (9.8)		
ABO blood type					0.37	0.47
Type A	28 (4)	3 (23)	25 (46)	46 (35)		
Type B	12 (18)	2 (15)	10 (18)	27 (20)		
Type O	23 (34)	6 (46)	17 (31)	55 (41)		
Type AB	5 (7.4)	2 (15)	3 (5.5)	5 (3.8)		

Abbreviations: HBGA, Histo-blood group antigen; Se, wild-type of the *FUT2* gene; Le, wild-type of the *FUT3* gene.

*P_*1*_ statistical test between the case subjects with moderate diseases and those with severe diseases. *P_*2*_ statistical test between the case subjects and the control subjects.

^$^The secretors carried at least one wild-type (Se) allele or the silent mutation (C357T) in the *FUT2 gene*. The weak secretors carried a missense mutation (A385T) in at least one allele of the *FUT2* gene that led to an amino acid change of I129F. The single nucleotide polymorphisms of C571T and G849A are both nonsense mutations.

^#^Lewis-positive genotype indicates that at least one allele is wild-type (Le). Lewis-negative genotype indicates that both *FUT3* alleles harbor nonsense mutations including G508A or T1067A.

**Table 2:** Histo-blood group antigens associated with rotavirus infections in Taiwanese children analyzed by conditional logistic regression.

Factor	mOR	95% CI	*P* value
Secretor status			
Secretor genotype	28.6	2.94–277	0.004
Weak-secretor genotype	Referent	…	…
Lewis blood type			
Lewis-positive genotype	16.8	1.08–261	0.04
Lewis-negative genotype	Referent	…	…
ABO blood types			
Type A	1.46	0.67–3.20	0.98
Type B	1.15	0.44–3.04	0.52
Type AB	2.80	0.50–15.7	0.32
Type O	Referent	…	…

Abbreviations: mOR, matched odds ratio; CI, confidence interval.

Two SNPs, G508A and T1067A, were identified in the *FUT3* gene, and both were nonsense mutations that disrupted the function of the encoded protein. The case subjects had more Lewis-positive genotypes than control subjects (99% versus 90%, *P* = 0.03). In the multivariate analysis, the Lewis-positive genotype was associated with a 16.8-fold higher risk of rotavirus infection compared with the Lewis-negative genotype (*P* = 0.04, Table 2). The distribution of ABO blood types did not differ significantly between cases and controls (*P* = 0.47, Table 1).

### Factors associated with disease severity in children with rotavirus infection

Younger age and male gender were two potential factors associated with increased disease severity (higher Vesikari score) (*P* = 0.04 and *P* = 0.06, respectively). The ethnic groups, rotavirus immunization, secretor status, Lewis genotype and ABO types were not associated with the disease severity in this cohort (Table 3).

**Table 3:** Factors associated with the severity (Vesikari score) of rotavirus AGE in 68 Taiwanese children, analyzed with a generalized linear regression model.

Factor	Adjusted means of the Vesikari score (95% CI)	F value	*P* value
Age (months)	…	4.42	0.04
Gender		3.67	0.06
Male	12.2 (8.3–16.2)		
Female	10.9 (6.9–14.9)		
Ethnics		1.55	0.21
Aboriginal	12.1 (7.5–16.8)		
Southeast Asian mother	12.2 (7.6–16.8)		
Minnan	10.3 (6.5–14.1)		
Hakka	11.6 (7.6–15.7)		
Rotavirus immunization		1.07	0.31
Yes	12.1 (8.3–15.8)		
No	11.1 (6.8–15.4)		
Secretor status		1.26	0.27
Secretor genotype	10.1 (7.1–13.1)		
Weak-secretor genotype	13.1 (7.1–19.0)		
Lewis blood type		2.24	0.14
Lewis-negative genotype	9.6 (3.6–15.6)		
Lewis-positive genotype	13.5 (10.5–16.5)		
ABO blood types		0.98	0.41
Type A	12.3 (8.3–16.3)		
Type B	12.1 (7.9–16.3)		
Type AB	10.1 (5.7–14.6)		
Type O	11.8 (7.8–15.7)		

## Discussion

Results from the current study clearly demonstrated that the secretor genotype and the Lewis-positive genotype were two independent host genetic determinants associated with increased risk of moderate-to-severe rotavirus AGE in Taiwanese children, while the ABO blood type was not related to rotavirus infections. In another case-control study addressing the association between FUT2 secretor status and severe rotavirus AGE in children in the US, the non-secretor status was identified in 23% of healthy controls but in only 0.5% of 189 rotavirus-positive cases^12^. The multivariate analysis demonstrated that the non-secretor FUT2 polymorphism was associated with up to 98% protection against severe AGE caused by rotavirus in the US children. Contrary to the well documented non-secretor phenotype in Caucasian populations, the distribution of the secretor status in distinct Asian ethnic groups has not been comprehensively evaluated. In the current study, all of the case and control subjects were either weak secretors or secretors. There were no non-secretors in this cohort. Nevertheless, as seen in non-secretors in the Caucasian population, the weak secretor was an important protective factor, and the A385T missense mutation in *FUT2* that led to an amino acid change of I129F was associated with an approximately 28-fold reduced risk of or 96.5% protection against moderate-to-severe rotavirus infections. Taken together, the data consistently support the essential role of the secretor phenotype in mediating rotavirus infection in distinct ethnic populations.

Notably, the Lewis antigen can be either protective or a risk factor for AGE caused by different enteropathogens. One of our recent studies in the Taiwanese population demonstrated that the Lewis-positive genotype is a protective factor against norovirus gastroenteritis in Taiwanese children^13^. By contrast, children with a Lewis-positive genotype were clearly at increased risk of rotavirus gastroenteritis in the current study. The Lewis enzyme accounted for the addition of the Lewis epitope, the α1,4-fucosyl residue, to the H type 1 or H type 2 precursors of HBGAs, to present the Le^b^ or Le^y^ antigens, respectively, on the surface of the epithelial cells^14^. This observation suggested that the Lewis epitope is a mediating ligand for rotavirus infection but might not be necessary for, or even interfere with, the recognition of the HBGAs in the epithelial cells on the gut surface by the norovirus.

Until the study was accomplished, rotavirus vaccines were not included in the extended program on immunization in Taiwan but were only provided in the private market as self-pay vaccines. A previous hospital-based AGE surveillance study involving 2,810 children estimated a rotavirus coverage rate of 21.6% during 2009 and 2011^15^. In the current study, the coverage rate doubled, and 43.9% children were immunized with any dose of rotavirus vaccines during 2015 and 2016. The overall vaccine effectiveness against rotavirus infections requiring hospitalization reached approximately 90%. Unfortunately, the manufactures of the rotavirus vaccines were not collected in this study. We were therefore unable to calculate the respective effectiveness of the currently used rotavirus vaccine (Rotarix^®^ and Rotateq^®^). Nevertheless, the data were consistent with another study in Taiwan, which disclosed that both rotavirus vaccines had a more than 90% effectiveness against rotavirus AGE requiring hospitalization^16^.

With the generalized linear regression, we were able to analyze the factors associated with the severity of rotavirus AGE (Vesikari score). Unexpectedly, we did not identify any association between HBGAs and disease severity. Because all of the cases had been hospitalized for their diarrheal illness, it is likely that the Vesikari scoring system was not sensitive enough to differentiate the severity of rotavirus AGE in this setting. Further, the study was not primarily designed to evaluate this outcome, and the sample size may not be sufficient to provide adequate statistical power. Nevertheless, we did identify age as a significant factor associated with disease severity. Intriguingly, the mean age was greater in cases with severe diseases compared with those with moderate diseases (43.5 months versus 39.7 months, Supplementary Table 1), but the difference was not of statistical significance. Similar results disclosing a greater proportion of rotavirus etiology in the elder age group were also observed in a hospital-based AGE surveillance study^15^. This finding suggests that the rotavirus is a virulent agent that can cause severe disease even in older age groups. Children younger than 2 years of age remain the most vulnerable population to severe rotavirus gastroenteritis.

There were several limitations of the study. First, the genotypes of the rotavirus strains were not determined due to the unavailability of a majority of stool samples. This limitation prevented us from exploring the association between the HBGA types and specific rotavirus genotypes. During the same period of study, data from an ongoing national surveillance study of pediatric AGE disclosed that among 199 rotavirus strains, G3P8 was the most common type and accounted for 108 cases (54.3%), which was followed by type G1P8 (33 cases, 16.6%), type G2P4 (30 cases, 15.1%), type G9P8 (15 cases, 7.5%) and other types (8 cases, 4.0%) (Prof. Yhu Chering Huang, unpublished data). Our finding regarding genetic susceptibilities should be confined to these contemporarily and locally circulating genotypes. Second, the majority of the study subjects were Han-Taiwanese and Hakka-Taiwanese. The other minor groups were not sufficiently represented. The results may therefore not be generalized to minor ethnic groups such as aborigines and immigrants from Southeast Asia. Third, all case subjects were hospitalized children with acute diarrhea. Thus, our finding applied only to subjects with moderate-to-severe rotavirus AGE but not to all rotavirus infections in general. Fourth, the sample size was relatively small, which can compromise the precise estimates of the vaccine effectiveness and the associations between HBGAs and rotavirus AGE.

In conclusion, this case-control study identified that secretor genotype and Lewis genotype are two independent genetic factors associated with increased risk of moderate-to-severe rotavirus AGE in Taiwanese children. The disease can be effectively prevented by immunization with either of the two currently available rotavirus vaccines.

## Methods

### Ethic statements

The study was approved by the Institutional Review Boards (IRBs) of Chang Gung Memorial Hospital (CGMH), Linkou branch, in March 2015, which allowed the determinations of the HBGAs genotypes and review of the patients’ medical information. Written informed consent was obtained from the subjects and/or their legal guardians before enrollment. All experimental protocols were performed in accordance with the guidelines and regulations of the IRB of CGMH.

### Study subjects

The study was prospectively conducted in Chang Gung Memorial Hospital from March 01, 2015, to June 30, 2016. During this period, the rotavirus-infected cases were identified by monitoring the reception log of stool samples that were subjected to rotavirus VP6 antigen detection with an enzyme immunoassay kit (RIDASCREEN Rotavirus, R-Biopharm AG, Darmstadt, Germany) in the viral laboratory on a daily basis. Rotavirus-positive cases and their caregivers were contacted in the ward and invited to the study. Once a rotavirus-infected case was enrolled, two age- and gender-matched potential controls were searched for among outpatients and healthy infants in the well-baby clinic. Those who were healthy or had acute diseases other than AGE were invited to participate in the study. The potential control subjects and their caregivers were interviewed, and informed consent was obtained before enrollment.

### Collection of clinical information

A standardized questionnaire form was used to collect the clinical information including demographic data and manifestations of AGE of the study subjects (Supplementary Table 1). The clinical information was obtained by interviewing the parents or primary caregivers of the study subjects and by reviewing the medical records. The Vesikari score was calculated for each case subject and was used to evaluate the severity of AGE^17^. The data were digitized and cleaned before we proceeded with the statistical analysis.

### Determination of single nucleotide polymorphisms (SNPs) in the FUT2, FUT3 and ABO genes

Oral mucosa swab and/or saliva samples were collected from each of the study subjects. Extraction of the genomic DNA was performed from the samples using the Qiagen QIAamp DNA Mini Kit (Qiagen, North Rhine Westfalia, Germany), according to the manufacturer’s instructions. The genotyping of *FUT2* and *FUT3* was performed using the PCR-RFLP method as previously described^9, 18^. Briefly, for typing *FUT2*, a fragment of the *FUT2* gene was amplified, and an 1149-bp PCR product was digested by *Msl*I and *Dde*I restriction enzymes and analyzed by agarose gel electrophoresis. Nested PCR was performed to sample position 385, and the PCR products were cleaved by the *Xmn*I restriction enzyme. The subjects were defined as secretors if at least one *FUT2* allele was wild-type (Se) or harbored silent mutations. The subjects were defined as weak secretors if both *FUT2* alleles harbored missense mutations or if one allele harbored missense mutations and the other allele harbored nonsense mutations. If both *FUT2* alleles were carrying nonsense mutations, the subjects were considered non-secretors.

For typing *FUT3*, various coding regions of the *FUT3* gene were amplified from genomic DNA with different primers sets, which were described elsewhere^18^. The PCR products were digested with restriction enzymes corresponding to the different primer sets. The digested fragment was analyzed by gel electrophoresis, and the *FUT3* genotype was determined according to the patterns in the gel. For uncertain cases or cases without any of the mutations mentioned above, direct sequencing or subcloning followed by sequencing of the PCR products was performed. The Lewis antigen was considered positive if at least one allele of the *FUT3* gene was wild-type (Le). If both alleles harbored nonsense mutations, the Lewis antigen was considered negative.

ABO types were determined according to the polymorphisms of the ABO gene, as described elsewhere^19^.

### Statistical analysis

The data were analyzed using SPSS Statistics, version 22.0 (IBM Corp., Armonk, NY, USA). Student’s *t-*test was used to compare the difference in demographic information and clinical manifestations between the patients and controls. The chi-square test was used to analyze the distributions of the *FUT2, FUT3* and *ABO* groups in the study subjects and healthy controls. Multiple conditional logistic regression analysis was applied to calculate the vaccine effectiveness against the rotavirus and explore the genetic factors associated with rotavirus AGE. A generalized linear model was used to analyze the factors associated with disease severity (Vesikari score) in patients with rotavirus infections. Statistical significance was set at a *P* value < 0.05 in the tests.

## Electronic supplementary material

Supplementary Information
