# Supplementary material for: Genetic Susceptibility to Rotavirus Gastroenteritis and Vaccine Effectiveness in Taiwanese Children
Source: Sci Rep. 2017 Jul 25;7:6412. doi: 10.1038/s41598-017-06686-y (PMC5526899; doi:10.1038/s41598-017-06686-y)
Supplement: Supplementary file 1 — Supplementary Information [file 41598_2017_6686_MOESM1_ESM.pdf]

Title: Genetic Susceptibility to Rotavirus Gastroenteritis and Vaccine Effectiveness in Taiwanese Children

Ting-An Yang[1#], Ju-Yin Hou[1#], Yhu-Chering Huang,[1,2], Chih-Jung Chen[1,2*]

[1]College of Medicine, Chang Gung University, 333 Taoyuan, Taiwan,

[2]Division of Paediatric Infectious Diseases, Department of Paediatrics, Chang Gung Memorial Hospital, 333 Taoyuan, Taiwan

[#]These authors contributed equally to this work

Supplementary Table 1. Characteristics of children with rotavirus gastroenteritis of distinct severity and demographics of the healthy controls, 2013-2016, northern Taiwan

Factor	Cases			Controls (n=133)	P_1*	P_2*
	All (n=68)	Moderate (n=15)	Severe (n=53)			
Demographics						
Male gender (%)	36 (53)	6 (40)	30 (57)	72 (54)	0.26	0.87
Age, months	42.6 ± 31.6	39.7 ± 39.2	43.5 ± 23.5	39.0 ± 36.0	0.69	0.48
Body weight, kg	16.0 ± 14.2	13.7 ± 7.9	16.7 ± 15.5	15.3 ± 9.9	0.48	0.66
Breast feeding (%)	62 (91)	13 (87)	49 (93)	109 (82)	0.49	0.08
Rotavirus vaccination (%)	8 (12)	3 (20)	5 (9.4)	77 (58)	0.26	<0.001
Household member having diarrhea (%)	29 (43)	7 (47)	22 (42)	5 (3.8)	0.72	<0.001
Underlying disease						
Atopic dermatitis (%)	9 (13)	3 (20)	6 (11)	22 (17)	0.38	0.54

Asthma (%)	0 (0)	0 (0)	0 (0)	6 (4.5)	--	0.75
Allergy rhinitis (%)	3 (4.4)	1 (6.7)	2 (3.8)	12 (9.0)	0.63	0.24
Nephrotic syndromes (%)	0 (0)	0 (0)	0 (0)	1 (0.8)	--	0.47
Developmental delay (%)	0 (0)	0 (0)	0 (0)	1 (0.8)	--	0.47
Malignant solid tumor (%)	0 (0)	0 (0)	0 (0)	1 (0.8)	--	0.47
Clinical features						
Hospital stay, days		5.4 ± 2.5	4.7 ± 2.1		0.25	
Fever (%)		12 (80)	51 (96)		0.03	
Fever duration, days		2.1 ± 1.5	3.6 ± 3.0		0.07	
Vomiting (%)		9 (60)	52 (98)		<0.001	
Max no. of vomiting per day		3.5 ± 5.2	7.6 ± 6.5		0.03	
Vomiting duration, days		1.6 ± 2.0	2.6 ± 1.3		0.03	
Diarrhea (%)		11 (73)	53 (100)		<0.001	
Max no. of diarrhea per day		4.1 ± 5.3	6.8 ± 4.7		0.06	
Diarrhea duration, days		1.8 ± 1.4	3.7 ± 1.5		<0.001	

Bloody stool (%)	2 (13)	6 (11)	0.83
Convulsion (%)	1 (6.7)	3 (5.7)	0.88
Use of parenteral antibiotics (%)	5 (33)	11 (21)	0.31
Laboratory data			
White blood cell count (/µl)	12380 ± 7508	10049 ± 5075	0.17
Immature neutrophil count (%)	0.9 ± 1.9	0.9 ± 2.8	0.99
Hemoglobin (g/µl)	11.9 ± 1.4	12.6 ±1.1	0.03
C-reactive protein (mg/l)	12.9 ± 16.5	14.4 ± 35.8	0.87
Positive stool bacterial culture (%)	1 (6.7)	0 (0)	0.06

*P_1 statistical test between the case subjects with moderate diseases and those with severe diseases. *P_2 statistical test between the case subjects and the control subjects
